# Prevention and control of major non-communicable diseases in China from 1990 to 2009: results of a two-round Delphi survey

**DOI:** 10.3402/gha.v6i0.20004

**Published:** 2013-02-12

**Authors:** Jun Lv, Miao Liu, Yu Jiang, Li-Ming Li

**Affiliations:** 1 Department of Epidemiology and Biostatistics, Peking University Health Science Center, Beijing, China; 2 Department of Epidemiology, Institute of Geriatrics, Chinese PLA General Hospital, Beijing, China; 3 Chinese Academy of Medical Sciences and Peking Union Medical College, Beijing, China

**Keywords:** non-communicable diseases, prevention and control, Delphi survey, China, developing countries

## Abstract

**Objective:**

The aim of this study was to learn about the progress of the prevention and control of major non-communicable diseases (NCDs) in China from 1990 to 2009 and to determine what prevention and control gaps remain based on the opinions of a group of panellists.

**Design:**

Sixty-four panellists, who are members of the Subcommittee of the Non-Communicable Diseases, the Expert Committee on Disease Control and Prevention established by China's Ministry of Health in 2010, were invited to participate in an email-based, two-round Delphi survey. In each round, a structured questionnaire was given to participants, who were asked to rate the importance and practical implementation of items relevant to the prevention and control of NCDs over two periods, 1990–1999 and 2000–2009, on a 10-point scale.

**Results:**

Of the 64 panellists invited, 20 (31.3%) completed the first-round survey, and, of those, 14 (70.0%) completed the second-round survey. Of the 237 common variables in the two survey rounds, there were 161 (67.9%) and 209 (88.2%) with an interquartile range ≤2 in the first round and second round, respectively. These results indicated a better expert consensus in the second round. There were 92 items in total in the second round of the questionnaire, 88 (95.7%) of which had median importance rating scores of equal to or greater than 7.00. The median scores for the practical implementation items during 2000–2009 were greater than those for the 1990–1999 period. The results indicate improved implementation in the recent decade.

**Conclusions:**

China has made progress in the prevention and control of NCDs during the 21st century. However, these intuitive rating results indicate that there are still large action gaps in the fight against epidemic NCDs in China.

China is undergoing a rapid rise in the burden of non-communicable diseases (NCDs), with major adverse social, economic, and health outcomes (1, 2). Two major forces are responsible for the emergence of NCDs in China. One is the very rapid transition of China's population from a young to an ageing population (3). The other, even more powerful force, is the rapid increase in high-risk lifestyle behaviours (1). The early NCD-targeted prevention and control practises in China can be traced back to 1958, and most of them were based on the academic research interests of medical experts prior to the 1990s. It was not until 1994 that the Ministry of Health (MOH) of China established an Office of Non-Communicable Diseases, affiliated with the Department of Disease Control. This change indicates that the prevention and control of NCDs became a responsibility of the government.

In the most recent decade, some efforts to prevent and control NCDs have been made in China. However, the escalating NCD burden indicates that these efforts were less effective than expected. So far, few studies or papers have shown, in a comprehensive manner, what strategy and implementation gaps between Chinese practise and global strategies for the prevention and control of NCDs remain, e.g. specific strategies and measures already taken, health workforce, financial input and allocation, and multisectoral collaboration. Such information, when available, often scatters in pieces and lacks external validity for the country as a whole. Most introductory reviews on the situation in China are based on individual author points of view, with a few selectively cited references and related narrative conclusions. Such papers usually devote more words to the prevalence of NCDs and their risk factors in China than they do to introducing the efforts in the fight against NCDs. Understanding these questions is important as a basis for decision-making and future action plans. NCD prevention and control is a multifaceted topic that warrants a series of surveys and studies devoted to understanding its practical implementation. However, expert consensus can be a better source of evidence than the experience of a single individual, which is the alternative when there is no other evidence available (4).

To improve government decision-making, the MOH established the Expert Committee on Disease Control and Prevention (ECDCP) in 2010, in which one of nine subcommittees is on NCDs (5). The subcommittee members consist of national experts from various fields of medicine and public health. We used a two-round Delphi survey of the panellists from the Subcommittee of Non-Communicable Diseases (SNCDs) to investigate their opinions on: (1) how much progress was achieved from 1990 to 2009 in China in the prevention and control of major NCDs, including cardiovascular disease, cancer, type 2 diabetes, and chronic obstructive pulmonary disease (COPD), and (2) how much room for improvement in the prevention and control of NCDs in China remains. This survey was developed to be one of the subcommittee's activities. The result would be used to inform the governmental decision-making on action plans for the next stage.

## Methods

The Delphi survey is a well-established technique for gathering expert opinions. The survey aims to establish a consensus on the parameter values of interest through a series of structured questionnaires or rounds (6, 7). The Delphi technique offers a number of specific advantages (6, 8–12): (1) it allows experts who are in geographically distinct locations to participate over time, and as a result, it is more cost-effective than convening multiple face-to-face meetings; (2) it allows a range of individuals to express their opinion without time or group pressures and provides the opportunity to revise individual views in response to group trends; (3) the structured communication feature helps facilitate group consensus, or score stability, while avoiding interpersonal influences; and (4) the survey tends to conclude with a moderate perceived sense of closure and accomplishment among participants.

### Participants

The SNCDs of the ECDCP are further divided into eight expert panels: Comprehensive Prevention and Control of NCDs, Cardiovascular Disease, Cancer, Chronic Respiratory Diseases, Diabetes, Nutrition, Ageing, and Oral Health. There are a total of 65 panellists in the SNCDs, and 64 of the 65 panellists were invited for participation. One of the authors was a panellist and therefore did not participate. All panellists were fully informed about the aims of the survey and consenting by taking part in the Delphi process.

### Instruments

The structured questionnaire was developed by researchers based on their rich practical experience in the prevention and control of NCDs in China and on their reading and understanding of many global strategic documents and published literature concerning the prevention and control of NCDs since the start of the 21st century. To avoid communication misunderstandings, the questionnaire was read by people not included in the study, which led to clarification and revision of the questionnaire.

The first-round questionnaire included four sections. The first section was designed to learn about the basic demographic and professional characteristics of the panellists. The second section included 83 questionnaire items covering seven aspects of NCD prevention and control, including: (A) scientific studies on NCDs (nine items); (B) general practises of prevention and control (nine items); (C) specific strategies and measures (30 items); (D) size and capability of workforce (four items); (E) financial input and allocation (12 items); (F) plans, strategies, and guidelines (10 items); and (G) roles in and responsibilities for NCD prevention and control among different government sector and multisector participation and collaboration (nine items). In this section, all panellists were asked to rate two aspects of each item. (1) How important is the content of the item given that the aim is to control the rapid rise of NCDs in China and reduce the heavy burden of NCDs on the individual, family, and society? Each item was rated in terms of importance from 1 (least important) to 10 (most important). (2) What was the practical implementation of the content of the item over two periods, namely, 1990–1999 and 2000–2009? Each item was rated in terms of its practical implementation from 1 (worst) to 10 (best). In the third section, the panellists were asked to self-rate their levels of expertise on the aforementioned seven aspects from 1 to 10 (lowest self-rated expertise to highest self-rated expertise) and the basis for their judgment. The bases for judgment included theoretical analyses, practical experiences, understanding of international and domestic professional peers, and intuition. The panellists were also asked to rate how large the impact of each basis for judgment was on their evaluation of items from 1 to 3 (small, moderate, and large, respectively). In the fourth section, space was left to allow the panellists to add additional items and to provide additional comments regarding the obstacles to implementing effective NCD prevention and control and suggestions for future development.

The second-round questionnaire included only the second and third sections of the first-round questionnaire. The results from the first round were used to revise the second section of the questionnaire. A few items were dropped, added, or revised in the second-round questionnaire. In the second round, the participants were provided with both their own scores and the mean group scores from the first round and were asked to reevaluate each item. All added and revised items were marked with a different colour font.

### Delphi process

Further rounds of survey would be more likely to lead to lower response rates than better estimates. Thus, we only conducted two rounds of the Delphi survey. All electronic instruments were sent via e-mail, including a questionnaire along with a cover letter that included an explanation of the study and the rating scales. The questionnaires were completed quasi-anonymously by the panellists; that is, the panellists knew the identities of some or all of the other individuals but did not know how they individually responded to any of the questions in any round. At most, two reminders were sent during each round in cases of non-response. The first-round survey was undertaken between December 28, 2010, and February 7, 2011, and the second round took place between February 22, 2011, and March 10, 2011. The second-round survey involved only the participants who completed and returned their first-round questionnaire to the researcher.

### Data analysis

The authority level of the panellists could indicate the possible level of confidence in the specific field of interest and help reflect the validity of the study to a certain extent (see supplemental file 1: The calculation and result of expert authority coefficient). For each item in the second section of the questionnaire, interquartile ranges (IQRs) and median scores were calculated as measures of dispersion and measures of central tendency, respectively. The IQR represents the distance between the 25th and 75th percentile values. It is generally accepted as an objective and rigorous method of determining consensus. As a rule of thumb, consensus is assumed to have been achieved when the IQR is no larger than two units on a 10-unit scale (13). In addition, we calculated the Kendall's W coefficient of concordance (W), which measured the agreement among the respondents in their overall ranking. This statistic ranges from 0 (no agreement) to 1 (perfect agreement). The agreement and confidence associated with the W coefficient can be interpreted as follows: 0.1, very weak agreement (no confidence in ranks); 0.3, weak (low); 0.5, moderate (fair); 0.7, strong (high); and 0.9, unusually strong (very high). All analyses were performed using PASW Statistics 18 (Release 18.0.0) (14).

## Results

Of the 64 subcommittee panellists invited to participate in the first round of the Delphi survey, 20 panellists (31.3%) completed the first-round questionnaire. Four additional panellists respond to our invitation. Two of them declined and gave the reason for not participating in this survey as lacking the expertise necessary to finish this questionnaire. One of them only filled out the fourth section of the questionnaire and provided some comments about the prevention and control of NCDs in China. Another one only rated the importance of the content of items in the second section. Forty panellists did not respond to our invitation after two reminders were sent. Of the 20 panellists who completed the first-round questionnaire, the median age was 52.5 years (P25, P75: 47.0, 57.0), and 17 participants (85.0%) were men. The median number of years they had spent in their current main specialty was 26.5 years (P25, P75: 22.0, 32.0). In the second round of the Delphi survey, 20 panellists who completed the first-round questionnaire were invited to participate in the second round. Of the 20 panellists who completed the first-round questionnaire, 14 (70.0%) returned the second-round questionnaire. Table 1 lists the size of each expert panel of the subcommittee, the number of participants, and the response rate of each expert panel in each round. The average authority coefficients were 0.76 and 0.77 for the first and second rounds, respectively (see supplemental file 1: The calculation and result of expert authority coefficient). The authority coefficient for scientific studies, practises, strategies, and measures, as well as for plans, strategies, and guidelines, was higher than those of the other three areas.

**Table 1:** The response rates of a two-round Delphi survey by expert panellists

		Number of participants	
		
Expert panels	Size of the panel (*N*)	First-round n1 (%)	Second-round n2 (%^b^)
Comprehensive Prevention and Control of NCDs	7^a^	2 (28.6)	2 (100.0)
Cardiovascular Disease	8	3 (37.5)	1 (33.3)
Cancer	9	2 (22.2)	1 (50.0)
Chronic Respiratory Diseases	7	1 (14.3)	1 (100.0)
Diabetes	8	2 (25.0)	0 (0.0)
Nutrition	8	5 (62.5)	4 (80.0)
Ageing	5	3 (60.0)	3 (100.0)
Oral Health	12	2 (16.7)	2 (100.0)
Total	64	20 (31.3)	14 (70.0)

a One of the seven panel members was one of the survey researchers and did not participate in the survey.

b The denominator for the response rate in the second round is the number of respondents in the first round.

Based on the feedback from the panellists in the first round, we developed several revisions for the instrument. A summary of the panellists’ ratings of the importance and practical implementation of each item in 1990–1999 and 2000–2009 in the second round is presented from Table 2 to Table 5. Of the 237 common variables (i.e. 79 items and three variables of each item evaluating the importance and practical implementation in the two periods) in the two rounds of the survey, there were 161 (67.9%) first-round variables with an IQR≤2, which increased to 209 (88.2%) in the second round. These results indicate a better consensus of panellists in the second round. The Kendall's W coefficient of concordance was 0.658 for the first round and 0.730 for the second round. There was strong agreement among the panellists in the second round. There were 92 items in total in the second round of the questionnaire, in which the median importance rating scores were less than 7.00 for four items (4.3%), ranged from 7.00 to 8.99 for 65 items (70.7%), greater than 9.00 for 25 items (25.0%). The median practical implementation score ratings for the 1990–1999 period were less than 4.00 for 35 items (38.0%), ranged from 4.00 to 4.99 for 42 items (45.7%), ranged from 5.00 to 5.99 for 12 items (13.0%), and were greater than 6.00 for three items (3.3%). The median practical implementation scores for the 2000–2009 period increased, which indicated improved implementation in the most recent decade. The median scores for the 2000–2009 period were less than 4.00 for 0 items (0.0%), ranged from 4.00 to 4.99 for 22 items (23.9%), ranged from 5.00 to 5.99 for 34 items (37.0%), and were greater than 6.00 for 36 items (39.1%).

**Table 2:** Summary of panellists’ ratings of the scientific studies on NCDs in 1990–1999 and 2000–2009 in the second round (*N*=14)

			Practical implementation	
			
	Median (IQR) score	Importance	Y1990–1999	Y2000–2009
	*By research content*			
A1	On the morbidity and mortality frequency of disease	9.00 (1.25)	5.00 (2.25)	7.00 (1.00)
A2	On the prevalence of disease risk factors	9.00 (1.25)	5.00 (2.25)	7.00 (0.50)
A3	On the aetiology of and risk factor for disease	8.00 (1.25)	4.00 (3.00)	6.00 (2.00)
A4	On the disease diagnosis	7.00 (1.25)	5.00 (1.00)	6.50 (2.00)
A5	On the disease treatment	8.00 (2.00)	5.50 (1.00)	7.00 (1.25)
A6	On the disease rehabilitation	7.00 (1.00)	4.00 (1.50)	5.00 (1.25)
A7	On the lifestyle intervention	9.00 (1.00)	5.00 (1.00)	7.00 (2.00)
A8	On the public policy	8.00 (1.25)	4.00 (2.50)	6.00 (2.00)
A9	On the economic evaluation	8.00 (1.00)	3.00 (2.50)	5.00 (2.00)
	*By disciplinary nature*			
A10*	Basic research	8.00 (1.50)	5.00 (2.00)	6.00 (0.75)
A11*	Clinical research	8.00 (2.50)	5.00 (2.00)	6.00 (1.75)
A12*	Prevention research	8.00 (1.50)	5.00 (3.00)	6.00 (2.50)
A13*	Translational research	8.00 (1.50)	4.00 (3.00)	5.00 (1.75)

* Items added or revised in the second round.

**Table 3:** Summary of panellists’ ratings of the practise in NCDs prevention and control in 1990–1999 and 2000–2009 in the second round (*N*=14)

			Practical implementation	
			
	Median (IQR) score	Importance	Y1990–1999	Y2000–2009
	*Of major NCDs*			
B1	Cancer	8.00 (1.50)	6.00 (1.50)	7.00 (1.00)
B2	Heart disease	9.00 (2.00)	6.00 (2.00)	8.00 (1.00)
B3	Cerebrovascular disease	9.00 (2.00)	6.00 (1.50)	7.00 (2.50)
B4	Type 2 diabetes	9.00 (1.50)	5.00 (1.00)	7.00 (1.50)
B5	Chronic obstructive pulmonary disease	8.00 (2.25)	5.00 (1.00)	6.00 (1.50)
	*Of major risk factors*			
B6	Tobacco use	9.00 (2.25)	4.00 (1.50)	6.00 (1.50)
B7	Unhealthy diet	8.00 (1.50)	4.00 (1.00)	6.00 (1.00)
B8	Physical inactivity	8.00 (1.50)	4.00 (2.00)	5.00 (1.00)
B9	Harmful use of alcohol	7.00 (2.00)	4.00 (2.00)	5.00 (1.50)
	*Surveillance of*			
C1	Morbidity	8.50 (1.25)	4.00 (1.25)	5.00 (1.00)
C2	Mortality	8.50 (2.25)	5.00 (1.25)	6.00 (2.00)
C3	Behavioural risk factors	8.00 (1.50)	4.00 (1.25)	5.00 (1.00)
	*Settings for health communication*			
C4	Healthcare facilities	8.00 (2.00)	4.50 (3.00)	6.00 (3.00)
C5	Schools	8.50 (2.00)	4.00 (1.50)	6.00 (1.00)
C6	Workplaces	8.00 (1.25)	4.00 (1.00)	5.00 (1.25)
C7	Community	9.00 (2.00)	4.00 (2.25)	6.00 (1.00)
C8	Other public places	7.00 (2.00)	3.00 (2.50)	5.00 (1.00)
	*Target population for health communication*			
C9	Elderly adults	8.00 (2.50)	4.50 (1.50)	6.00 (2.00)
C10	Young adults	8.00 (1.25)	4.00 (2.00)	5.00 (1.25)
C11	Youth	9.00 (2.00)	4.00 (2.00)	6.00 (2.50)
	*Channels for health communication*			
C12	Dissemination of, e.g. leaflets, brochures, and posters	6.00 (1.25)	4.50 (1.00)	6.00 (1.25)
C13	Community health lectures	7.00 (1.25)	4.00 (1.25)	6.00 (1.25)
C14	Theme-day campaign	6.50 (2.00)	4.00 (1.25)	6.00 (1.25)
C15	Mass media channels	9.00 (1.00)	4.00 (1.25)	6.00 (3.00)
C16	Doctor-patient communication	8.00 (2.25)	4.00 (2.00)	6.00 (2.00)
	*Contents for health communication*			
C17	On diseases	7.50 (2.00)	4.50 (1.00)	6.00 (1.25)
C18	On lifestyle risk factors	8.00 (1.25)	4.00 (1.25)	6.00 (1.00)
C19	Economic evaluation of health communication programmes	8.00 (1.00)	3.00 (1.25)	4.00 (1.00)
C20	Educational messages in line with current best-available evidence	8.00 (0.25)	4.00 (2.25)	5.00 (1.00)
	*Screening programmes*			
C21	Screening for disease	8.50 (2.00)	4.00 (2.00)	6.00 (1.25)
C22	Providing effective diagnosis, treatment and intervention for individuals identified through early screening	8.00 (1.25)	3.50 (1.25)	5.00 (2.00)
C23	Effectiveness evaluation of screening programmes	8.00 (1.50)	4.00 (1.00)	5.00 (1.50)
C24	Economic evaluation of screening programmes	7.00 (1.00)	4.00 (1.50)	4.00 (2.00)
	*Intervention, treatment, management, and rehabilitation*			
C25	Intervention for high-risk populations	8.50 (1.25)	4.00 (1.00)	4.00 (1.25)
C26	Treatment of patients	8.00 (2.25)	5.00 (1.25)	5.50 (2.25)
C27	Rehabilitation of patients	7.50 (1.50)	3.00 (2.00)	4.00 (2.00)
C28	Patient self-management	8.00 (1.25)	4.00 (2.00)	4.00 (2.50)
C29	Treatment plan in line with current best-available evidence	8.00 (2.25)	4.50 (2.25)	4.00 (3.25)
C30	Standardisation of treatment and management	8.50 (1.25)	3.00 (1.50)	4.00 (3.25)

**Table 4:** Summary of panellists’ ratings of the health workforce and finance in 1990–1999 and 2000–2009 in the second round (*N*=14)

			Practical implementation	
			
	Median (IQR) score	Importance	Y1990–1999	Y2000–2009
	*Adequate number of staff working on the prevention and control of NCDs*			
D1*	In urban healthcare facilities	8.00 (1.75)	3.00 (2.00)	4.50 (2.75)
D2*	In rural healthcare facilities	8.00 (1.00)	2.50 (1.75)	4.00 (2.50)
D3*	In public health facilities	8.00 (1.75)	3.00 (2.00)	4.50 (2.75)
	*Well-trained staff with capabilities of*			
D4	Scientific research	8.00 (1.00)	4.00 (2.00)	5.50 (1.25)
D5	Practise in NCD prevention and control	8.50 (1.00)	4.00 (2.25)	5.00 (1.00)
D6	Leadership	8.00 (0.00)	4.00 (2.00)	5.00 (2.00)
	*Providing regular training for staff in*			
D7*	Scientific research	7.00 (1.00)	3.00 (1.75)	4.50 (2.50)
D8*	The practise of NCD prevention and control	7.50 (1.75)	3.00 (2.00)	4.50 (2.75)
D9*	Leadership	7.00 (2.00)	3.00 (1.75)	5.00 (1.75)
	*Input*			
E1	Regular funds from central government	9.00 (1.25)	2.50 (1.00)	5.00 (1.00)
E2	Regular funds from local government	9.00 (1.25)	2.00 (1.00)	4.00 (1.25)
E3	Specific funds for particular programmes of government ministries	8.00 (1.25)	3.00 (1.50)	5.00 (1.00)
E4*	Other sources of funds	6.50 (2.50)	3.00 (2.00)	5.00 (2.50)
	*Allocation to*			
E5	Scientific research	6.50 (2.00)	4.00 (2.00)	5.00 (1.75)
E6	Lifestyle intervention	8.00 (0.50)	3.00 (1.00)	5.00 (0.00)
E7	Screening for disease	8.00 (0.50)	3.00 (1.00)	5.00 (0.25)
E8	Treatment of patients	7.00 (2.00)	4.00 (1.50)	5.00 (1.00)
E9	Rehabilitation of patients	7.50 (1.25)	3.00 (1.00)	5.00 (1.25)
E10	Economic evaluation of NCD prevention and control programmes	7.00 (1.25)	2.50 (1.25)	4.00 (2.00)

* Items added or revised in the second round.

**Table 5:** Summary of panellists’ ratings of the national policy and multisector collaboration in 1990–1999 and 2000–2009 in the second round (*N*=14)

			Practical implementation	
			
	Median (IQR) score	Importance	Y1990–1999	Y2000–2009
	*Plans*			
F1	Development	8.00 (1.50)	4.00 (2.00)	6.00 (1.00)
F2	Implementation	9.00 (1.50)	3.00 (2.00)	5.00 (1.00)
	*Standard operating procedure*			
F3*	Development	9.00 (0.75)	3.00 (2.00)	5.00 (2.00)
F4*	Implementation	9.00 (2.50)	2.00 (1.00)	4.00 (2.00)
	*Public policies*			
F5	Development	8.50 (2.00)	3.00 (2.00)	4.50 (1.00)
F6	Implementation	9.00 (2.00)	3.00 (2.00)	4.00 (1.00)
	*Guidelines*			
F7	Development	9.00 (1.25)	3.50 (2.00)	6.00 (2.00)
F8	In line with current best-available evidence	9.00 (1.00)	4.00 (2.00)	6.00 (1.25)
F9	Applicable to Chinese population	9.00 (2.00)	4.00 (2.00)	5.50 (1.00)
F10	Practical and operational	9.00 (1.25)	3.50 (1.00)	5.50 (1.25)
F11	Training for professionals to improve adherence to guidelines	9.00 (1.25)	4.00 (1.25)	5.00 (0.25)
F12	Practical implementation	8.00 (1.25)	3.00 (2.00)	5.00 (2.00)
	*With a clear definition of roles and responsibilities in NCD prevention and control*			
G1	Public health facilities	8.00 (1.00)	3.50 (1.50)	5.00 (1.00)
G2	Healthcare facilities	8.00 (1.50)	3.00 (1.25)	5.00 (1.00)
G3	Non-healthcare sectors	7.00 (2.25)	2.50 (1.25)	4.00 (0.50)
	*Actual involvement in NCD prevention and control*			
G4	Public health facilities	9.00 (1.00)	4.00 (2.25)	6.00 (1.00)
G5	Healthcare facilities	9.00 (1.25)	4.00 (2.00)	6.00 (1.25)
	*Actual involvement of non-healthcare sectors in NCD prevention and control*			
G6	Establishment of effective coordination mechanisms between healthcare sectors and non-healthcare sectors	8.00 (1.25)	3.00 (2.00)	4.00 (1.00)
G7	Involvement of non-healthcare sectors in the campaigns or programmes for NCD prevention and control initiated by healthcare sectors	8.00 (2.00)	3.00 (1.25)	4.00 (2.00)
G8	Healthcare sector involvement in the policy-making and decision-making of non-healthcare sectors	8.00 (1.50)	3.00 (1.50)	4.00 (1.00)
G9	Policy and actual operation of non-healthcare sectors supporting healthy lifestyles, or at least not promoting unhealthy lifestyles	8.00 (1.50)	2.00 (1.00)	4.00 (2.00)

* Items added or revised in the second round.

Based on ratings of panellists, scientific research on NCDs (item A series) has grown rapidly in China in recent years. In contrast, the areas of economic evaluation, disease rehabilitation, public policy, and disease aetiology were somewhat lagging behind other research areas. Basic research and disease-oriented clinical research was better developed than population-based prevention research and translational research.

The practises of prevention and control of major NCDs in general was scored higher than those of major risk factors (item B series). The surveillance of mortality was better developed than those of morbidity and behavioural risk factors (items C1–C3). The workplaces and young adults were less covered by the health communication programme (items C4–C11). The channels for health communication, such as disseminating posters, giving health lectures in the community, and initiating theme-day campaigns, were rated to be less important by panellists but better developed than mass-media channels and doctor-patient communication (items C12–C16). In addition, in intervention programmes, including health communication and screening, there has been the lack of a rigorous programme evaluation of their impact and effectiveness, especially their economic costs and benefits (items C19 and C24).

The national workforce for the prevention and control of NCDs was inadequate in size, especially in rural areas (items D1–D3). The workforce research capability was scored better than the capabilities of practise and leadership (items D4–D6). The specific funds for particular programmes of various government ministries had always played a more important role than other sources of funds in the prevention and control of NCDs in China (items E1–E4), which indicates a programme-based financing mechanism.

The panellists agreed that the last decade has seen some progress in the development of national plans and public policies for NCD prevention and control, but their implementation has been far less than expected. (item F series). The low ratings for items G6–G9 by panellists also reflect that there has been no effective intersectoral coordination mechanism in existence.

## Discussion

We utilised a Delphi survey to intuitively and quantitatively verify that China has made progress in the prevention and control of NCDs in the most recent decade compared to the 1990–1999 period. However, there are still large gaps in the efforts against the NCD epidemic in China. To our knowledge, this is the first study to attempt to depict, in a comprehensive manner, the extents of both the efforts to fight NCDs in China over the past 20 years and the efforts that will be needed in the near future.

The evidence generated from epidemiological surveillance plays a very important role in the policy-making process. There are two mortality data systems currently in operation in China: the Ministry of Health–Vital Registration (MOH-VR) system and the Diseases Surveillance Points (DSP) system (15). A Chronic Disease Risk Factor Surveillance in Chinese adults was established by the CCDC in 2004 in the form of a series of cross-sectional surveys carried out every three years (i.e. 2004, 2007, and 2010) that use a multistage cluster sampling design based on the DSP system (16). For NCD incidence evaluation, most of the ongoing surveillance focuses on several major chronic diseases, such as ischemic heart disease, cerebrovascular disease, diabetes mellitus, and cancer, and is programme-based or developed voluntarily at the city or district (or county) level instead of at the national level. In brief, mortality surveillance of NCDs in China is more systematic and well developed than behavioural risk factor and incidence surveillance. Meanwhile, the quality of the NCD surveillance system is uneven across different regions (17).

National intervention demonstration programmes for the prevention and control of NCDs have increased since 2000 (18). Overall, these programmes have been mostly disease-centred and have used high-risk approaches such as early screening and case management. In contrast, the intervention activities, such as the Healthy Lifestyle Initiative (2007–2015) (19), that involve common behavioural risk factors (e.g. smoking, unhealthy diet, and physical inactivity) as their focus are limited and insufficient. If we look at local Centres for Disease Control and Prevention (CDCs), which are the main bodies in charge of the implementation of population-based intervention activities for NCD prevention and control in China, only 93.5, 51.5, and 30.8% of provincial-, city-, and county-level CDCs, respectively, implemented intervention activities in 2008 (17). Furthermore, most of the intervention activities were programme-based instead of being part of CDCs’ routine work. In other words, various programmes include only the cities or districts (counties) that are willing to commit to a programme with wise leaders and competent, motivated teams at their field or demonstration sites. However, it gradually becomes a vicious cycle of ‘all or nothing’ and underlies large regional disparities regarding the prevention and control of NCDs. The evidence and experience required for success are always unlikely to be copied to other regions due to political commitment limitations, available resources, and the capability and motivation of the teams. Another major problem with these intervention programmes has been the lack of a rigorous programme evaluation of their impact and effectiveness, especially their economic costs and benefits.

Health communication based on a superficial population-wide approach (20) is the most commonly employed approach for disease prevention and control (17). The channels for health communication, such as disseminating posters, giving health lectures in the community, and initiating theme-day campaigns, were rated to be less important by panellists but better developed than mass-media channels and doctor-patient communication in reality. Settings such as schools and communities are more easily accessed through local education authorities and street-level administrative authorities. There is not an easy way to access the private workplace. Owing to the limitations of the settings, the channels, and the health communication contents, young adults free of chronic disease received less benefits from such intervention activities than the elderly or patients.

It was shown in our results that the national workforce for the prevention and control of NCDs was inadequate in size. For example, there were 7,483 CDC personnel working in NCD prevention and control in 2008, which only accounted for 4.0% of all staff (17). The average numbers of staff working on the prevention and control of NCDs were 9.5, 3.5, and 2.2 at the provincial, city, and county level CDCs, respectively (17). In other words, one worker in the CDC system worked for an average of 175,000 (min, max: 40,000, 342,000) people in China (17). The better workforce research capability compared to capabilities of practise and leadership may be partially attributable to programme-based financing mechanisms and funding- and paper-oriented performance evaluation mechanisms.

Adequate investment is crucial for the sustainable prevention and control of NCDs. Although there has been a significant increase in regular funds from the central government for sustained prevention and control practises on a regular basis, investment remains inadequate, especially from local governments. Taking the regular funds of the CDC system for NCD prevention and control as an example, the proportions of CDCs that had regular funds in 2008 were 100.0, 71.7, and 71.0% at the provincial, city, and county level CDCs, respectively (17). Specifically, the average annual funds for NCD prevention and control (the percentage accounting for the total funds of the CDCs) in 2008 were approximately USD 137,000 (2.29%), USD 14,000 (1.70%), and USD 7,000 (2.69%) at the provincial, city, and county level CDCs, respectively (17). Therefore, the prevention and control of NCDs in China is significantly underfunded.

The health and quality of life of individuals and populations are determined by a complex set of interrelated factors, and the choices, even for individual lifestyles, are influenced by forces that are outside the control of individuals, especially children (21). Such complexity requires multisectoral action to address the NCD epidemic. However, the intervention activities for the prevention and control of NCDs have always been confined to the healthcare sector alone in China. Although the multisectoral strategy can be clearly observed in recent plans and policies, the roles and responsibilities of non-healthcare sectors in the prevention and control of NCDs remain to be clarified. However, it is worth pointing out that China actually had a remarkable model of the multisectoral action for health, namely the Patriotic Health Campaign (22). It has led to great success in improving sanitation, hygiene, and communicable disease control and is exactly what the efforts aimed at NDC prevention and control should use for reference.

In this study, we provided a panel of experts with a predesigned questionnaire for their ratings and used the Delphi technique to obtain their consensus opinion. More than 95% of items were rated 7 or greater for their importance related to the prevention and control of NCDs. These results indicate that these questions were reasonably relevant to our topic. This Delphi survey was halted after two rounds, and more than 88% of items reached consensus using IQR≤2 as the cutoff. A greater number of rounds would potentially lead to a minor increase in convergence, but the falloff in response rate definitely reduces their usefulness. The diversity of opinions on several items may reflect genuine uncertainty and strikingly diverse views.

A low response rate is a common limitation of Delphi-based studies and also a major concern for the validity of our results. The composition of expert panellists and their response rate results indicate that some expert panellists with limited specialties, such as oral health, chronic respiratory diseases, cancer, and diabetes, tended to have lower response rates. About four-fifths of panellists who did not respond are clinical experts and had a patient-focused daily work. In contrast, half of the responders have a population-focused daily work that enables them to be more likely to understand the bigger picture. These panellists were invited based on their membership in the SNCDs of the ECDCP instead of their capability of providing expert opinion on the situation of prevention and control of NCDs in general. Lacking of expertise on these general issues might be a main reason of preventing them from responding to our survey. The average authority coefficient of 0.77 suggested that these results from the panellists who responded to our survey can be treated with high levels of confidence. If we asked each expert to respond regardless of fact that they lacked the expertise necessary to finish the questionnaire to achieve a high response rate, the authority level of these results will be reduced. The authority coefficients concerning financial input and allocation, the functions and multisector actions, and the health professional workforce were lower than those in the other four areas. It is unlikely that a higher response rate would result in a significant improvement because the majority of panellists represent a specific specialty of medicine, public health, and work location (hospitals, national and provincial CDCs, universities, and research institutes). The reality is that there was a lack of expertise in finance, human resources, and multisectoral coordination among this panel of experts. Despite the potential bias introduced by non-response, these results are consistent with our general understanding of these issues.

This study provides us with a unique opportunity to learn not only about the historical development and present status of the prevention and control of NCDs in China but also about the consensus opinions of a group of panellists on this issue. These panellists were selected based on their membership in the SNCDs of the ECDCP. As they have the opportunity to express their professional opinions and suggestions, the panellists play a critical role in influencing top policy decisions regarding the prevention and control of NCDs in China, especially at the present stage of lacking more rigorous evidence. Although additional surveys and studies will be needed to understand the challenges associated with practical policy implementation, this study of expert consensus is the first step toward informed decision-making.

## Conclusions

Facing a growing burden of NCDs, China has made progress in the prevention and control of NCDs since the beginning of the 21st century. However, the rating results from this study indicate that there are still large gaps in the efforts to fight against the epidemic of NCDs in China. The coming 10 years are a critical time for China in the prevention and control of the threat posed to the country's prosperity by the growing burden of NCDs (2). High priority should be given to the management of NCDs instead of focusing too much on other pressing health issues. Sustainable mechanisms should be developed instead of programme-based prevention practises. The United Nations’ high-level meeting on NCD prevention and control in 2011 provided a powerful outside force for Chinese government to address the challenge of NCD.
